# Supplementary material for: A bacterial checkpoint protein for ribosome assembly moonlights as an essential metabolite-proofreading enzyme
Source: Nat Commun. 2019 Apr 4;10:1526. doi: 10.1038/s41467-019-09508-z (PMC6449344; doi:10.1038/s41467-019-09508-z)
Supplement: Supplementary file 1 — Supplementary Information [file 41467_2019_9508_MOESM1_ESM.pdf]

Supplementary Information

A bacterial checkpoint protein for ribosome assembly moonlights as an essential metabolite-proofreading enzyme

A. J. Sachla and J. D. Helmann

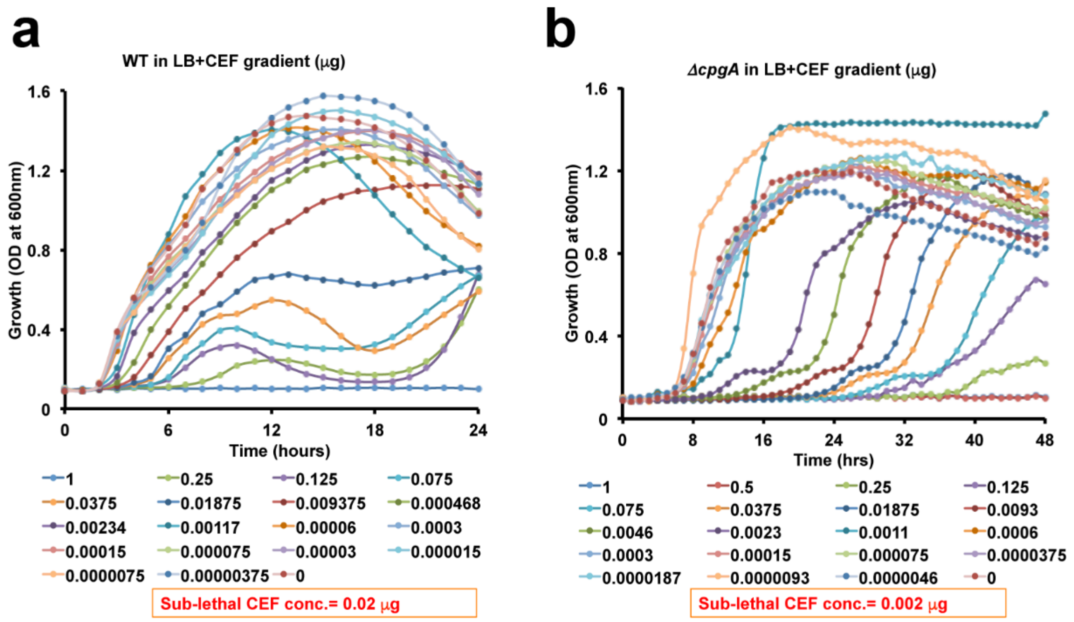

Supplementary Figure 1: Δ*cpgA* cells are hypersensitive to CEF. Growth of (a) WT168 and (b) Δ*cpgA* tested in the presence of varying amounts (0-1) μg gradient of CEF tested in 200 μl of LB at 37 °C aerobically by monitoring OD at 600 nm. The sub-lethal concentration value was defined as the concentration of CEF that reduced growth by half as judged by an OD_{600} <0.8 at 12th hr (for WT) and OD_{600} <0.8 at 18th hr (for Δ*cpgA*). The data are representative of 2 independent biological replicates.

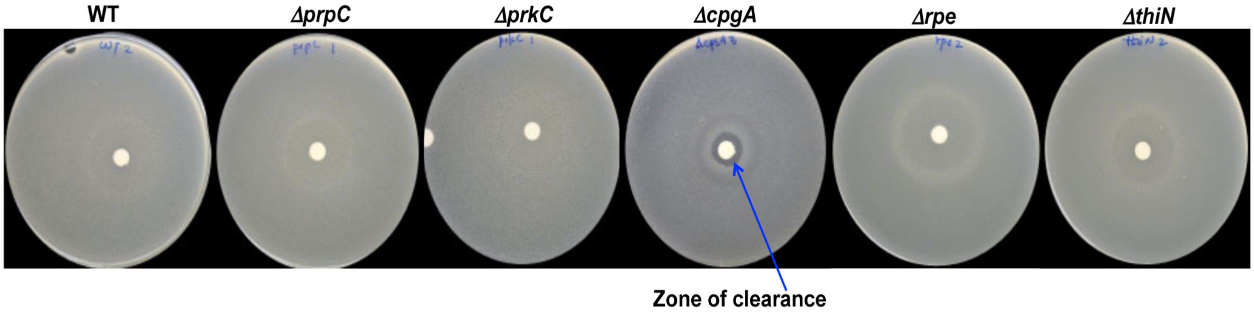

Supplementary Figure 2: Glucose toxicity is exclusively due to loss of *cpgA*. Disk diffusion assays using glucose (55 μmol) with a blue arrow indicating the clearance zone for Δ*cpgA* cells. Cells were grown on gluconeogenic, MH medium for strains with deletions of upstream (*prpC*-phosphatase and *prkC*-PASTA kinase) and downstream (*rpe*-ribulose-5-phosphate epimerase and *thiN*-thiamine pyrophosphokinase) genes relative to *cpgA*.

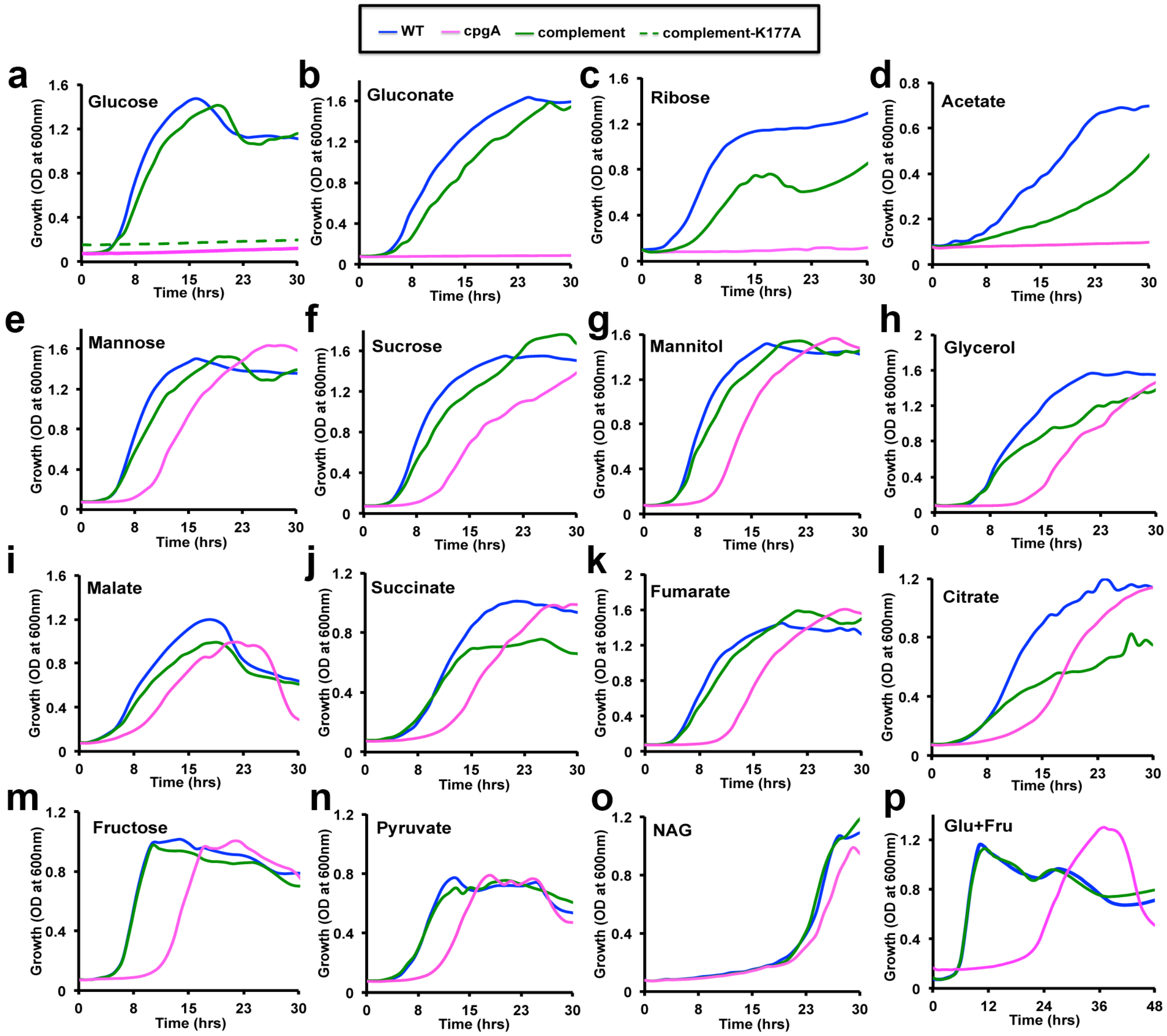

Supplementary Figure 3: Δ*cpgA* is defective in utilizing glycolytic and PPP carbon sources. Growth monitored in MSMM supplemented with 0.8% of (a) glucose, (b) gluconate, (c) ribose, (d) acetate, (e) mannose, (f) sucrose, (g) mannitol, (h) glycerol, (i) malate, (j) succinate, (k) fumarate, (l) citrate, (m) fructose, (n) pyruvate, (o) NAG (0.3 %), and (p) glucose plus fructose (measured for 48 hrs) for WT (blue line), Δ*cpgA* (pink line) and complement (green line) strains. For panel (a), the dashed line shows the inability of CgpA with an active site mutation to complement. Data are representative of at least three independent biological replicates.

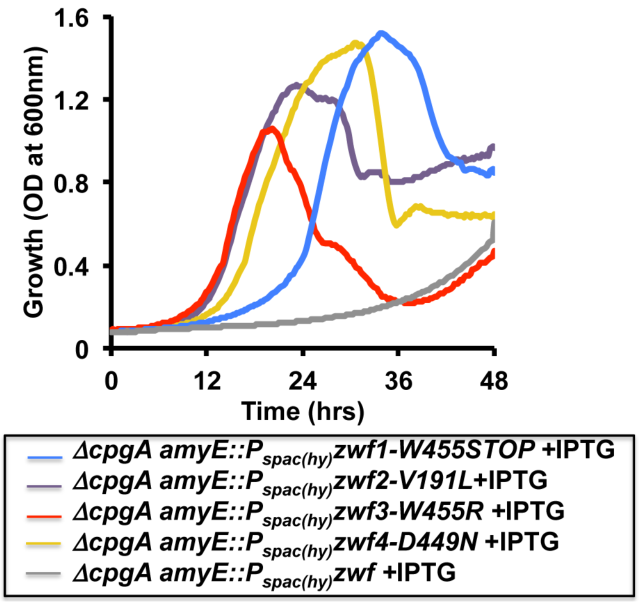

Supplementary Figure 4: A reduction of Zwf activity can overcome glucose sensitivity of Δ*cpgA*. Growth for Δ*cpgA* carrying *amyE::P*$_{spac(hy)}$*zwf* (grey line), *amyE::P*$_{spac(hy)}$*zwf1-W455stop* (blue line), *amyE::P*$_{spac(hy)}$*zwf2-V191L* (purple), *amyE::P*$_{spac(hy)}$*zwf3-W455R* (red line), and *amyE::P*$_{spac(hy)}$*zwf4-D449N* (yellow line) strains was monitored in MSMM with 0.8% glucose with 0.5 mM IPTG at 37 °C for 48 hours. Data are representative of three different biological replicates. Since these strains contain wild-type *zwf* at the native locus, the restoration of growth with mutant alleles suggests a dominant effect.

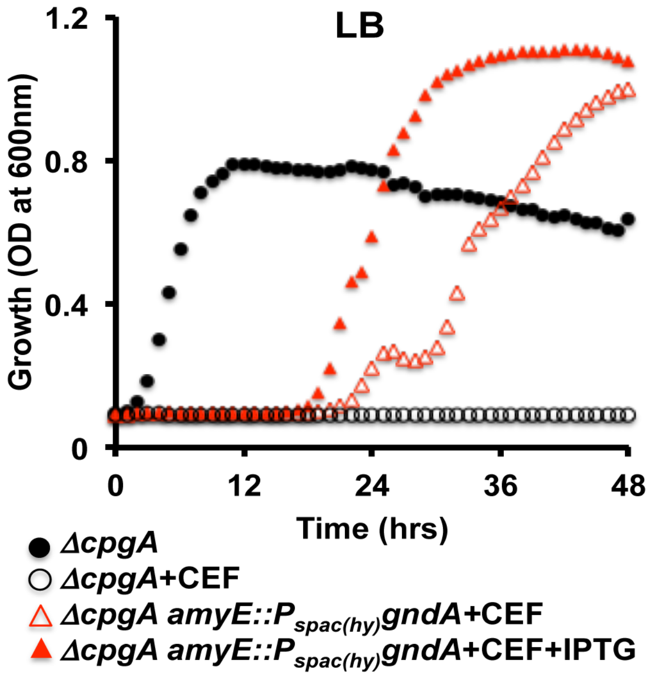

Supplementary Figure 5: GndA expression circumvents 6-phosphogluconate accumulations and leads to CEF resistance. Growth in LB medium (200 µl) at 37 °C of Δ*cpgA* (filled black circles) is completely inhibited with 0.1 µg CEF (open black circles). However, growth with 0.1 µg CEF is restored by expression of GndA (Δ*cpgA amyE::P*$_{spac(hy)}$*gndA*-open triangle), especially in the presence of IPTG (filled triangle). The data are representative of 3 independent biological experiments.

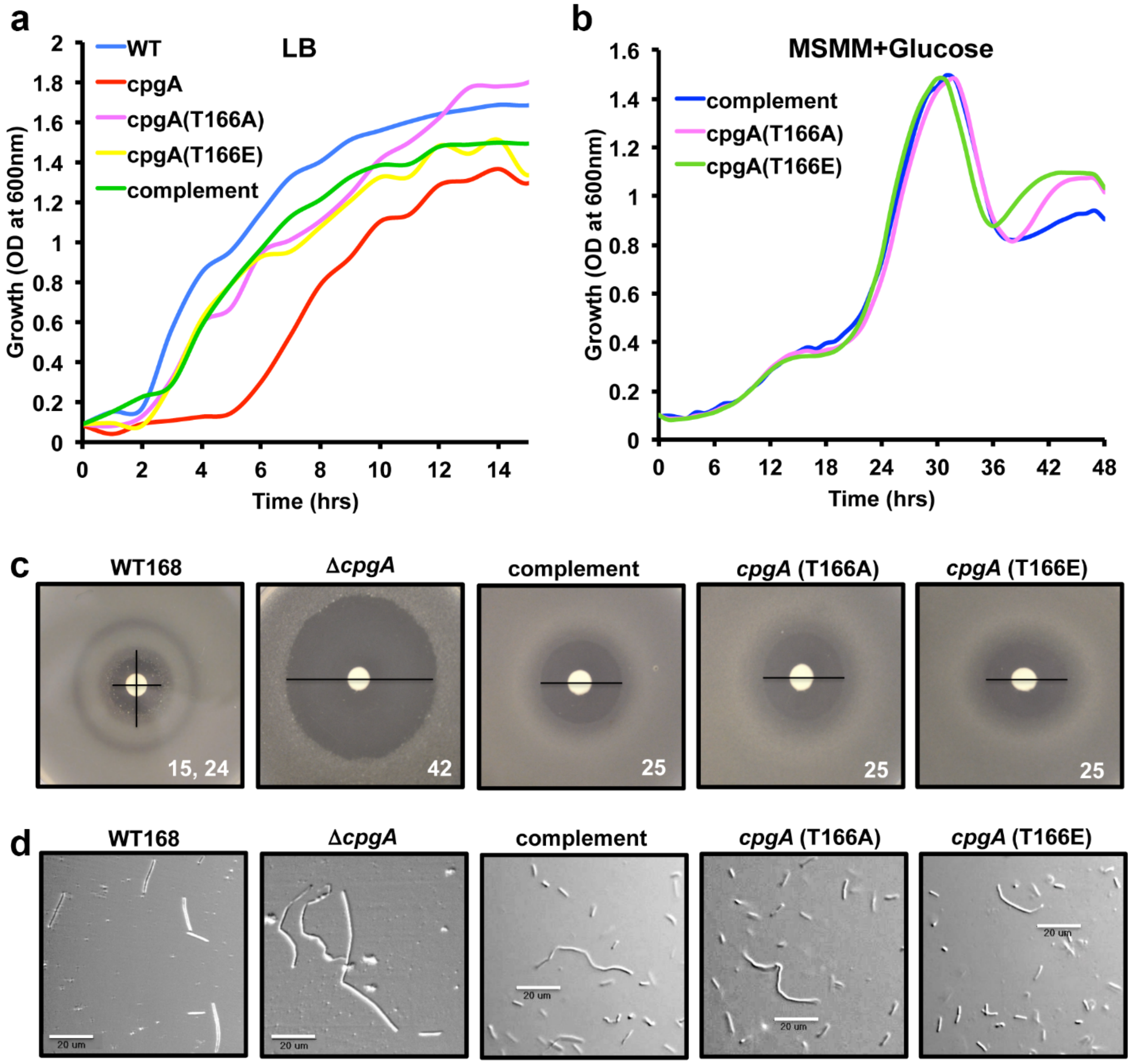

Supplementary Figure 6: The metabolite-proofreading role of CpgA is independent of phosphorylation by PrkC. (a) Growth curves for WT (blue line), Δ*cpgA* (red line), complemented strain (green line), and Δ*cpgA* expressing phospho-replacement mutations at Thr166 [A-ablative (pink line) or E-mimetic (yellow line)] of CpgA tested in LB medium. (b) Growth comparison of phospho-replaced CpgA [T166A (pink line) or E (green line)] with wild-type complement (blue line) in MSMM with 0.8% glucose. (c) CEF sensitivity (6 μg) assessed by disk diffusion assay on LB media. The measured diameter of clearance (indicated with black lines) is enumerated in mm (white font). (d) Evaluation of cell morphology (LB, mid-exponential phase) using differential phase contrast microscopy, scale bar (grey line) is 20 μm.

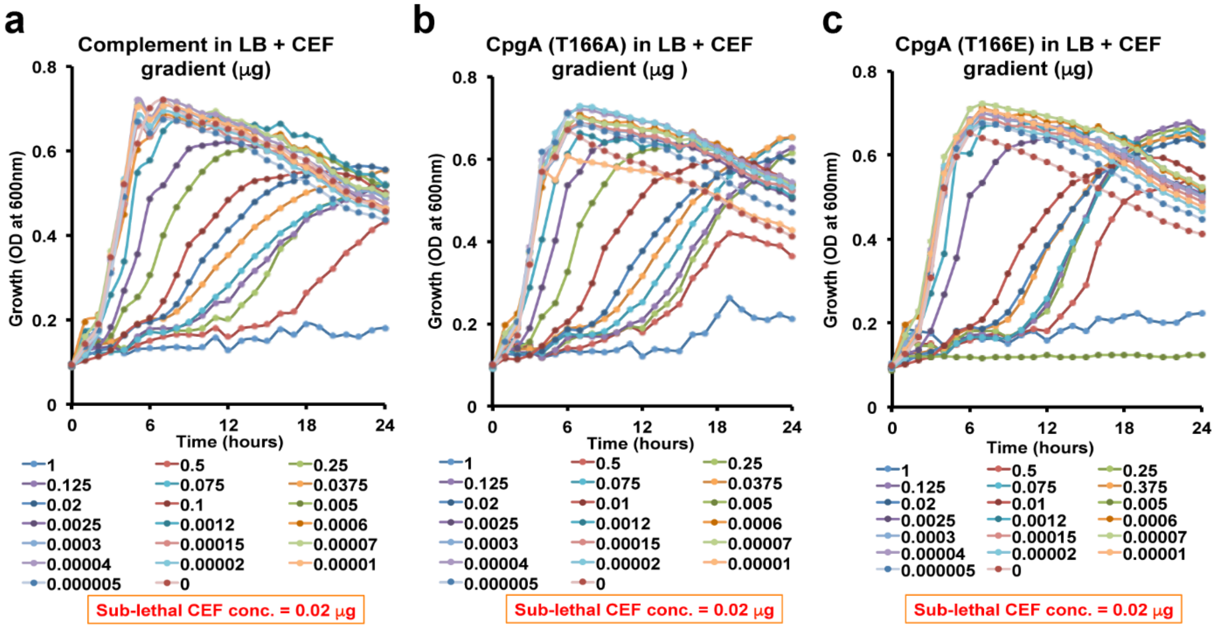

Supplementary Figure 7: Phosphorylation at Thr166 of CpgA is not required for CEF resistance. The growth of Δ*cpgA* cells upon re-introduction of CpgA **(a)**, T166A-phosphoablative **(b)**, and T166E-phosphomimetic **(c)** CpgA versions in the presence of varying concentrations of CEF (0-1 μg) monitored in LB broth at 37 °C for 24 hrs in 0.1 ml of final volume. The sub-lethal levels were defined as the CEF concentration that reduced growth to <0.4 OD_{600} at 10th hr. The data are representative of 2 independent biological replicates.

Supplementary Table 1: List of Suppressor Mutations

Strain	Gene (allele)	Nucleotide change	Amino acid change	Reference position	Gene function
**mTn insertions for CEF selection**					
ΔcpgA.1	*ptsG*	n/a	n/a	1458515	PTS system glucose-specific transporter subunit IICBA
ΔcpgA.2	*yfnI*	n/a	n/a	796100	minor lipoteichoic acid synthetase
ΔcpgA.3	*rho*	n/a	n/a	3803816	transcription terminator factor
ΔcpgA.4	*trmNF-yazA*	n/a	n/a	43602	tRNA1(Val)(adenine(37)-N6)-methyltransferase
**Glucose selection**					
ΔcpgA.5	*zwf1*	G-->A	Trp455*	2480519	Glucose-6-phosphate dehydrogenase of pentose phosphate pathway
ΔcpgA.6	*zwf2*	G-->T	Val191Leu	2479726	Glucose-6-phosphate dehydrogenase of pentose phosphate pathway
ΔcpgA.7	*zwf3*	T-->C	Trp455Arg	2480518	Glucose-6-phosphate dehydrogenase of pentose phosphate pathway
ΔcpgA.8	*zwf4*	G-->A	Asp449Asn	2480500	Glucose-6-phosphate dehydrogenase of pentose phosphate pathway
**Gluconate selection**					
ΔcpgA.9	*gntP*	G-->A	Trp351*	4116762	Gluconate uptake
**Glucose plus gluconate selection**					
ΔcpgA.10	*zwf5*	G-->A	Gly46Arg	2479291	Glucose-6-phosphate dehydrogenase of pentose phosphate pathway
	yodE	T deletion	Thr35fs	2129891	dioxygenase/glyoxalase
ΔcpgA.11	*zwf6*	G-->A	Trp455*	2480489	Glucose-6-phosphate dehydrogenase of pentose phosphate pathway
	ptsH	G-->A	Gly54Asp	1459544	Histidine containing phosphocarrier protein of PTS system
ΔcpgA.12	*yhjO*	C-->T	Gly34Arg	1133285	putative multidrug transporter
ΔcpgA.13	*zwf7*	AAAGCTTGGCGG deletion	Lys392-Gly395 deletion	2480328	Glucose-6-phosphate dehydrogenase of pentose phosphate pathway
	ppsD	G-->A	Ala382Val	1973712	plipastatin synthetase
ΔcpgA.14	*zwf8*	G-->A	Val191Met	2479726	Glucose-6-phosphate dehydrogenase of pentose phosphate pathway
	zwf9	ATT-->TAG	Ile192*	2479729	
	zwf10	A-->G		2479734	
	zwf11	T insertion	Ala195fs	2479739	
ΔcpgA.15	*zwf12*	T-->G	Trp33*	2479254	Glucose-6-phosphate dehydrogenase of pentose phosphate pathway
ΔcpgA.16	*zwf13*	C-->T	Arg23*	2479222	Glucose-6-phosphate dehydrogenase of pentose phosphate pathway
	fliH	G-->A	Gly181Ser	1695794	flagilar assembly protein, negative regulator of FliI ATPase
ΔcpgA.17	*zwf14*	G-->T	Trp203Leu	2479763	Glucose-6-phosphate dehydrogenase of pentose phosphate pathway
ΔcpgA.18	*zwf15*	G-->A	Ala118Thr	2479507	Glucose-6-phosphate dehydrogenase of pentose phosphate pathway
ΔcpgA.19	*zwf16*	AACC deletion	Gln5fs	2479165	Glucose-6-phosphate dehydrogenase of pentose phosphate pathway
	yebD-yebE	C-->T	n/a	697427	Intergenic region betweenyebD and yebE
ΔcpgA.20	*zwf17*	CACT insertion	Arg274fs	2479970	Glucose-6-phosphate dehydrogenase of pentose phosphate pathway
ΔcpgA.21	*zwf18*	G-->A	Asp238Asn	2479861	Glucose-6-phosphate dehydrogenase of pentose phosphate pathway
ΔcpgA.22	*zwf19*	G-->A	Gly46Arg	2479291	Glucose-6-phosphate dehydrogenase of pentose phosphate pathway
	yodE	T deletion	Thr35fs	2129891	dioxygenase/glyoxalase
**Glucose plus gluconate selection**					
ΔcpgA.23 amyE::P_{spac} PHO13	trnSL-Ala1	G-->C	n/a	3194466	Alanine tRNA, information processing in translation
ΔcpgA.24 amyE::P_{spac} PHO13	yetA	C-->T	n/a	778753	unknown
ΔcpgA.25 amyE::P_{spac} PHO13	frr	G-->A	n/a	1720528	ribosome recycling factor
ΔcpgA.26 amyE::P_{spac} PHO13	dhbF	T-->C	n/a	3282367	involved in 2,3-dihydroxybenzoate biosynthesis (bacillibactin synthesis)
ΔcpgA.27 amyE::P_{spac} PHO13	yfjO	G-->A	Gly258Arg	874173	unknown but similar to RNA methyltransferase
**STOP codon*					

Supplementary Table 2: Strains used in this study

Strain	Genotype	Construction	Reference
B. subtilis			
168	*trpC2*	Lab strain	Lab stock
HB20401	*trpC2 cpgA ::erm*	BGSC	Lab stock
HB20476	*trpC2 prpC ::erm*	BGSC	Lab stock
HB20451	*trpC2 prkC ::erm*	BGSC	Lab stock
HB20667	*trpC2 rpe ::erm*	BGSC	Lab stock
HB20666	*trpC2 thiN ::erm*	BGSC	Lab stock
HB20007	*trpC2 ΔprpC*	pDR244-->HB0467	This study
Hb20006	*trpC2 ΔprkC*	pDR244-->HB20451	This study
HB20409	*trpC2 ΔcpgA*	pDR244-->HB20401	This study
HB20422	*trpC2 prpCprkC ::erm*	PCR fusion HB20476+HB20451 -->168	This study
HB20467	*trpC2 ΔprpCprkC*	pDR244-->HB20422	This study
HB20423	*trpC2 prkCcpgA ::erm*	PCR fusion HB20401+HB20451 -->168	This study
HB20538	*trpC2 ΔprkCcpgA*	pDR244-->HB20423	This study
HB21661	*trpC2 ΔprpC::erm ΔcpgA*	gDNAHB20476-->HB20409	This study
HB20424	*trpC2 prpCprkCcpgA ::erm*	PCR fusion on HB20476+HB20401-->168	This study
HB20477	*trpC2 ΔprpCprkCcpgA*	pDR244-->HB20424	This study
HB20686	*trpC2 ΔcpgA ::cpgA-pMUTIN4 (erm)*	cpgA-pMUTIN4-->HB20409	This study
HB20429	*trpC2 ΔcpgA pMarA (erm)*	pMarA-->HB20409	This study
HB20503	*trpC2 ptsG ::erm*	BGSC	Lab stock
HB21564	*trpC2 yazA ::erm*	BGSC	Lab stock
HB21565	*trpC2 yfnI ::erm*	BGSC	Lab stock
HB20505	*trpC2 ΔptsG*	pDR244-->HB20503	This study
HB20532	*trpC2 ΔptsG cpgA ::erm*	gDNA HB20401-->HB20505	This study
HB20443	*trpC2 ΔcpgA rho ::spec*	gDNA rho::spec-->HB20409	This study
HB20440	*trpC2 ΔcpgA yazA ::erm*	gDNAHB21564-->HB20409	This study
HB20444	*trpC2 ΔcpgA yfnI ::erm*	gDNAHB21565-->HB20409	This study
HB20519	*trpC2 ΔcpgA amyE ::P* $_{spac(hy)}$ *-cpgA (cat)*	pPL82-*cpgA* -->HB20409	This study
HB21550	*trpC2 ΔcpgA amyE ::P* $_{spac(hy)}$ *-cpgA(K177A) (cat)*	pPL82-*cpgA* -->HB20409	This study
HB21549	*trpC2 ΔcpgA amyE ::P* $_{spac(hy)}$ *-pgi (cat)*	pPL82-*pgi* -->HB20409	This study
HB21550	*trpC2 ΔcpgA amyE ::P* $_{spac(hy)}$ *-zwf (cat)*	pPL82-*zwf* -->HB20409	This study
HB21551	*trpC2 ΔcpgA amyE ::P* $_{spac(hy)}$ *-ykgB (cat)*	pPL82-*ykgB* -->HB20409	This study
HB21552	*trpC2 ΔcpgA amyE ::P* $_{spac(hy)-}$ *gndA (cat)*	pPL82-*gndA* -->HB20409	This study
HB21553	*trpC2 ΔcpgA amyE ::P* $_{spac(hy)}$ *-gntZ (cat)*	pPL82-*gntZ* -->HB20409	This study
HB21554	*trpC2 ΔcpgA amyE ::P* $_{spac(hy)}$ *-gapA (cat)*	pPL82-gapA-->HB20409	This study
HB21555	*trpC2 ΔcpgA amyE ::P* $_{spac(hy)}$ *-aldolase (cat)*	pPL82-aldolase-->HB20409	This study
HB21556	*trpC2 ΔcpgA amyE ::P* $_{spac(hy)}$ *-eno (cat)*	pPL82-eno-->HB20409	This study
HB21557	*trpC2 ΔcpgA amyE ::P* $_{spac(hy)}$ *-pgk (cat)*	pPL82-*pgk* -->HB20409	This study
HB21558	*trpC2 ΔcpgA amyE::P* $_{spac(hy)}$ *-pgm (cat)*	pPL82-*pgm* -->HB20409	This study
HB20639	*trpC2 zwf ::erm*	BGSC	Lab stock
HB20683	*trpC2 ΔcpgA zwf ::erm*	gDNA HB20639-->HB20409	This study
HB21551	*trpC2 ΔcpgA amyE ::P* $_{spac(hy)}$ *-zwfV191L (cat)*	pPL82-*zwfV191L* -->HB20409	This study
HB21552	*trpC2 ΔcpgA amyE ::P* $_{spac(hy)}$ *-zwfD449N (cat)*	pPL82-*zwfD449N* -->HB20409	This study
HB21553	*trpC2 ΔcpgA amyE ::P* $_{spac(hy)}$ *-zwfW455R (cat)*	pPL82-*zwfW455R* -->HB20409	This study
HB21554	*trpC2 ΔcpgA amyE ::P* $_{spac(hy)}$ *-zwfW455STOP (cat)*	pPL82-*zwfW455STOP* -->HB20409	This study
HB21555	*trpC2 ΔcpgA zwf ::erm amyE ::P* $_{spac(hy)}$ *-zwf (cat)*	pPL82-zwf-->HB20683	This study
HB21556	*trpC2 ΔcpgA zwf ::erm amyE ::P* $_{spac(hy)}$ *-zwfW455STOP (cat)*	pPL82-zwfW455STOP-->HB20683	This study
HB21738	*trpC2 gntP ::erm*	BGSC	Lab stock
HB21739	*trpC2 gntK ::erm*	BGSC	Lab stock
HB21801	*trpC2 ΔcpgA gntP ::erm*	gDNA HB21738-->HB20409	This study
HB21802	*trpC2 ΔcpgA gntK ::erm*	gDNA HB21739-->HB20409	This study
HB24001	*trpC2 ΔcpgA amyE ::P* $_{spac(hy)}$ *-PHO13 (cat)*	pPL82-PHO13-->HB20401	This study
HB24204	*trpC2 cpgA::cpgA(T166A) (erm)*	LFH PCR (cpgAT166A-erm-rpe)-->HB20409	This study
HB24209	*trpC2 cpgA::cpgA(T166E) (erm)*	LFH PCR (cpgAT166E-erm-rpe)-->HB20409	This study
HB24211	*trpC2 cpgA::cpgA (erm)*	LFH PCR (cpgA-erm-rpe)-->HB20409	This study

Supplementary Table 3: Primers used in this study

Name	Sequence	Reference	Purpose
cpgAupF	ATGCGGGCACTGTCATTG	This study	
cpgAdownR	TCCGCCATTCGCTTAACCTC	This study	Deletion confirmation
cpgAInF	TTCTCAGCGGTTCAGC	This study	
cpgAInR	CGGTGTATCTGCAACC	This study	internal primers for deletion check
cpgAkpnIF	CGTAGGTACCATGCCTGAGGGCAAAATTATTAAG	This study	
cpgArPsiIR	ATATGGTACCATACCTCGGCTTTCTGTCTTTAATCTC	This study	pMUTIN4 based cloning
cpgAHindIIIF	CTGAAAGCTTAAAGGAGGAAGGATCAATGCCTGAGGGCAAAATTATTAAG	This study	
cpgAXbaIR	CAGTTCTAGACTAATACCTCGGCTTTCTGTC	This study	pPL82 based cloning
cpgAFT166E	ACATCATCCCGCATTTTCAGGATAAAGAAACGGTATTTGCCGGTCAGTCCGGTGTT	This study	
cpgART166E	AACACCGGACTGACCGGCAAATACCGTTTCTTTATCCTGAAAATGCGGGATGATGT	This study	Site-directed mutagenesis (LFH)
cpgAFT166A	CATCATCCCGCATTTTCAGGATAAAGCAACGGTATTTGCCGGTCAGTCCGGTGTT	This study	
cpgART166A	AACACCGGACTGACCGGCAAATACCGTTGCTTTATCCTGAAAATGCGGGATGATG	This study	Site-directed mutagenesis (LFH)
cpgAT177AF	CGGTATTTGCCGGTCAGTCCGGTGTTGGGGCATCCTCGCTTCTCAACGCGATCAGTG	This study	
cpgAT177AR	CGGACTGATCGCGTTGAGAAGCGAGGATGCCCCAACACCGGACTGACCGGCAAATA	This study	Site-directed mutagenesis
ptsGupF	TCCTGAGGAGTACCAAATTG	This study	
ptsGdownR	AGCTTTATCCTGACCCAG	This study	Deletion confirmation
PgiXbaIF	CAGTTCTAGAAAAGGAGGAAGGATCAATGACGCATGTACGCTTTGACTAC	This study	
PgiBglIIR	CGTTAGATCTTTAATCTTCCAGACGTTTTTCAAGCTC	This study	*pgi* clonining in pPL82
AldoXbaIF	ACTGTCTAGAAAAGGAGGAAGGATCAATGCCTTTAGTTTCTATGAC	This study	
AldoBglIIR	AAGTAGATCTTTAAGCTTGGTTTGAAGAACCAAATTC	This study	*fbaA* clonining in pPL82
GapAHindIIIF	CTGAAAGCTTAAAGGAGGAAGGATCAATGGCAGTAAAAGTCGGTATTAAC	This study	
GapAXbaIR	CAGTCTAGATTAAAGACCTTTTTTTGCGATG	This study	*gapA* cloning in pPL82
PgkHindIIIF	CTGAAAGCTTAAAGGAGGAAGGATCAATGAATAAAAAAACTCTCAAAGAC	This study	
PgkXbaIR	CTTTTCTAGATTATTTATCGTTCAGTGCAGCTAC	This study	*pgk* cloning in pPL82
PgmXbaIF	CAGTTCTAGAAAAGGAGGAAGGATCAAatgAGTAAAAAACCAGCTGCAC	This study	
PgmBglIIR	TATGAGATCTTTATTTTTGAATTAAAGATGTTCCTG	This study	*pgm* cloning in pPL82
EnoXbaIF	ACTGTCTAGAAAAGGAGGAAGGATCAATGCCATACATTGTTGATG	This study	
EnoBglIIR	CATGAGATCTTTACTTGTTTAAGTTGTAGAAAGAG	This study	*eno* cloning in pPL82
PykXbaIF	CAGTTCTAGAAAAGGAGGAAGGATCAAATGAGAAAAACTAAAATTGTTTG	This study	
PykBglIIR	CGTTAGATCTTTAAAGAACGCTCGCACGGCCTTG	This study	*pyk* cloning in pPL82
ZwfSF	AGCAGTAATTGTCATATTCGGTG	This study	
ZwfdownR	GAGGAAGAAGAGATTGAATTGTC	This study	Deletion confirmation
ZwfXbaIF	CAGTTCTAGAAAAGGAGGAAGGATCAAGTGAAAACAAACCAACAACCAAAAG	This study	
ZwfBglIIR	ATTTAGATCTTTATATGTTCCACCAGTGTAAGCCGTC	This study	*zwf* clonining pPL82
YkgBXbaIF	CAGTTCTAGAAAAGGAGGAAGGATCAATGACAAAATACATAGGATATGTGGGAAC	This study	
YkgBBglIIR	TATTGAGATCTTTATACTTGATGTAAAAACTTTACACAAAC	This study	*ykgB* cloning in pPL82
GndAXmaIF	ATATCCCGGGAAAGGAGGAAGGATCAATGTCAAAACAACAAATCGGCGTTATC	This study	
GndAClaIR	AAGGATCGATTTACTTCATCCATTCAGTATGGAAGATGC	This study	*gndA* cloning in pPL82
GntZXbaIF	CAGTTCTAGAAAAGGAGGAAGGATCAATGTTCAATTCGATTGGTGTCATAG	This study	
GntZBglIIR	TAAGAGATCTTTATTCAGACCAATTCGTATGGAAGAC	This study	*gntZ* cloning in pPL82
gntPupF	GAATCCGGATGTACGCGGCTC	This study	
gntPdownR	CTGCGATTTTCGTCAGGATAG	This study	Deletion confirmation
gntKupF	AATGGGAGCGGTCGTAATTGGTTTAAC	This study	
gntKdownR	GGAAACCGTGTGTGACGGATAGTG	This study	Deletion confirmation
marinerUpF	GCTTGTAAATTCTATCATAATTG	Lab stock	
marinerDownR	AGGGAATCATTTGAAGGTTGG	Lab stock	mTn insertion check
YeastPHO13XbaIF	CAGTTCTAGAAAAGGAGGAAGGATCAATGACTGCTCAACAAGGTGTAC	This study	
YeastPHO13BglIIR	CGTTAGATCTCTATAACTCATTATTGGTTAAGGTGTAG	This study	PHO13 cloning in pPL82
mlsF	GAATACGGGTTTGCTAAAAG	This study	phospho-replacement-LFH
mlsR	CTTTTAGCAAACCCGTTATTC	This study	phospho-replacement-LFH
rpeRdown	TCACCAGGAGGGAACATTTGAATATG	This study	phospho-replacement-LFH

*underlined nucleotide sequence represents the restriction sites for cloning
